# Integrated Phenolic, Volatilomic and Sensory Profiling of Two Finger Lime Cultivars and Faustrime Grown in a Mediterranean Region

**DOI:** 10.3390/foods15183266

**Published:** 2026-09-16

**Authors:** Giulia Mozzo, Cosimo Taiti, Maria Alfonsi, Giovanni Spinelli, Diego Comparini, Bruno Bighignoli, Marta Beccaluva, Stefano Mancuso, Elisa Masi

**Affiliations:** 1 Department of Agriculture, Food, Environment and Forestry (DAGRI), University of Florence, Viale Delle Idee 30, Sesto F.No, 50019 Florence, Italyelisa.masi@unifi.it (E.M.); 2 Group of Agroecology, Centre of Plant Sciences, Sant’Anna School of Advanced Studies, 56127 Pisa, Italy; 3 Department of Biology, Università di Firenze, via Micheli 3, 50121 Firenze, Italy

**Keywords:** *Citrus australasica*, Faustrime, citrus caviar, fruit quality, total phenolic content, PTR-ToF-MS

## Abstract

Finger lime (*Citrus australasica*) is an Australian native citrus valued as a “citrus caviar” in high-end gastronomy, and its cultivation is expanding in the Mediterranean region. Comparative quality data for different genotypes grown under Italian conditions are still scarce. This study characterised two finger lime cultivars, ‘Pink Ice’ (FPI) and ‘Rainforest Pearl’ (FRF), and the trigeneric hybrid Faustrime (FAU), cultivated in Central Italy and harvested at commercial maturity, through an integrated assessment of morphometric, physicochemical, histochemical and sensory traits. The three accessions were clearly differentiated. Faustrime produced the largest and heaviest fruits and the largest juice vesicles, whereas Rainforest Pearl fruit was the smallest. All accessions showed high titratable acidity (8.93–11.56% citric acid) and low sugar content, with Rainforest Pearl reaching the highest sugar concentrations. Pink Ice presented the highest total phenolic content in the directly consumed pulp (919.09 mg GAE/kg FW), while Rainforest Pearl showed the greatest accumulation of terpenoids in the peel, revealed by Nadi histochemical staining and confocal microscopy. Volatile compounds, profiled by PTR-ToF-MS, and sensory evaluation further separated the accessions, with Faustrime marked by a pronounced citronella aroma. These findings provide a quality baseline for cultivation of finger lime and Faustrime in a Mediterranean region during the 2024 season, and support genotype-specific market positioning according to sensory, nutritional and aromatic characteristics.

## 1. Introduction

Across Europe, demand for a wide range of exotic fruits has grown steadily in recent years. This trend is driven by increasing consumer awareness of the health benefits associated with fruit consumption, a growing interest in diversified diets, the expansion of ethnic communities, and greater exposure to international food cultures through travel and global communication [1,2]. Interest in emerging exotic fruits market has steadily increased among the European market [3]. However, a considerable number of fresh and processed tropical fruit products remain relatively unknown within European markets. Finger lime (*Citrus australasica*) is an Australian native citrus species that has recently attracted growing worldwide interest as a high value “citrus caviar” for gastronomy and as a potential functional food ingredient [4,5]. Its distinctive finger-shaped fruit, brightly coloured juice vesicles, and intense aromatic profile make it an attractive premium specialty crop, distinguishing it from more conventional citrus fruits [6,7]. In parallel with the continued expansion of global citrus demand, native Australian citrus species are increasingly regarded as promising novelty crops and valuable sources of innovative products for both fresh consumption and processing industries. Finger lime has rapidly evolved from a wild species into a niche crop of international commercial interest, with commercial orchards established in Australia [8,9]. High quality fruits are currently supplied to markets in the European Union, Asia, the United Kingdom, Canada, and Japan, where the fresh, caviar-like juice vesicles are primarily used as an edible garnish or to impart flavour and texture to culinary preparations, particularly in gourmet cuisine and mixology. Finger lime fruits are also used for the production of value-added products such as jams, sauces, beverages, and dried peel [10]. Besides its culinary appeal, finger lime has attracted increasing scientific and industrial interest owing to its high content of bioactive phenolic compounds and its reported antioxidant and anti-inflammatory properties [11,12]. More recently, commercial plantings and experimental trials have been established outside Australia, including in the United States, China, Brazil, Japan, and several European countries [8,13]. Despite its increasing commercial relevance, finger lime remains largely unfamiliar to most consumers outside niche gastronomic markets. Consumer acceptance and willingness to purchase exotic fruits like finger lime largely depend on how familiar they are with the fruit [14,15]. While visual appeal and perceived value are important, the fruit’s flavour and aroma must meet consumer expectations. If the sensory experience does not meet the high expectations posed by the fruit’s unique appearance and price, it can lead to disappointment and reduce the likelihood of repeat purchases [16].

Consequently, ensuring a consistent and high-quality sensory profile is crucial for the successful market development of this emerging crop. Alongside finger lime, Faustrime (Citrus × faustrimedin), a trigeneric hybrid derived from *C. australasica*, *C. aurantiifolia*, and *Fortunella japonica*, has also attracted increasing interest in commercial cultivation. In Italy, Faustrime is frequently grown alongside finger lime cultivars and represents a potentially valuable complementary crop. However, its distinct genetic background results in markedly different chemical and volatile profiles compared with those of finger lime, as reported for fruits cultivated in Central Italy [16]. Both finger lime and Faustrime are premium specialty citrus fruits whose market value depends heavily on sensory quality and consumer perception. Because these fruits do not ripen further after harvest, choosing the right time to pick them is crucial for ensuring the best quality, especially since factors like taste, aroma, and bioactive compounds are key to their appeal [17,18]. Previous studies have shown that secondary metabolites, including terpenoids and polyphenols, undergo significant changes under Mediterranean conditions [19]. Nevertheless, despite the documented influence of genotype on fruit composition, the final quality of finger lime and Faustrime fruits is determined by the complex interaction among genotype, environment, and harvest stage [11].

Fruit quality is a multidimensional trait resulting from the interaction among morphological, physicochemical, metabolic, aromatic and sensory characteristics. Although these attributes are often investigated separately, their integration provides a more comprehensive description of cultivar identity and commercial value. Integrated fruit quality fingerprinting, combining complementary analytical approaches, has successfully discriminated high-value horticultural products and identified cultivar-specific quality traits [20].

Earlier studies comparing finger lime cultivars from Australia and Florida found that phytochemical composition varies substantially depending on the cultivar. Johnson et al. [13] found that pulp ascorbic acid content ranged from 23 to 54 mg/100 g and total phenolic content from 144 to 197 mg/100 g across five Australian cultivars, while Adhikari et al. [11] reported that Florida selections with red pulp consistently had higher levels of phenolics, flavonoids, and anthocyanins, as well as greater antioxidant capacity, compared to selections with pale or white pulp. However, these studies focused only on single trait categories, such as phytochemical or volatile composition, without accompanying morphometric, histochemical, or sensory data. Furthermore, it is still unclear to what extent different genotypes of finger lime cultivars and related hybrids differ in fruit morphology, physicochemical traits, sensory qualities, and volatile composition. Such information is essential for supporting cultivar selection, optimizing harvest management, and enhancing the commercial valorisation of these emerging specialty citrus fruits.

Therefore, the present study aimed to establish an integrated fruit quality fingerprint of two finger lime cultivars (‘Pink Ice’ and ‘Rainforest Pearl’) and the related hybrid Faustrime, cultivated in Central Italy, by combining morphometric, physicochemical, histochemical, volatilomic and sensory analyses.

## 2. Materials and Methods

### 2.1. Plant Material

The plant material used in this study was obtained from Società Agricola Jucci Santoro, located in the Municipality of Sezze (LT), Central Italy (41.452201 N, 13.101190 E). During the April–October 2024 growing period, the mean minimum and maximum air temperatures were 15.1 and 27.5 °C, respectively, while cumulative precipitation was 403.8 mm [21]. The experiment was conducted in October 2024 using hand-harvested fruits of two finger lime (*Citrus australasica* F. Muell.) cultivars, ‘Rainforest Pearl’ (FRF) and ‘Pink Ice’ (FPI), together with Faustrime (FAU). Fruits were collected on 10 October, corresponding to the onset of the commercial harvest season for finger lime under Mediterranean growing conditions and to the beginning of fruit maturity [19]. Eighteen fruits per genotype were randomly sampled at commercial maturity the upper, middle, and lower canopy positions of each tree, to account for within-tree variability. After collection, the fruits were transported to the laboratory and stored in a climate-controlled chamber at 5 °C until analysis. Six fruits were used for morpho-physical characterization and subsequently allocated to chemical analyses: three were used for HPLC and titratable acidity analyses, while the remaining three were used for total soluble solids (°Brix) and total polyphenol determination. Three additional fruits were used for both confocal microscopy and PTR-ToF-MS (Ionicon Analytik GmbH, Innsbruck, Austria) analyses, whereas the remaining nine fruits were used for sensory evaluation by a panel of ten assessors.

### 2.2. Morphochemical Analyses of the Fruits

#### 2.2.1. Weight, Flavedo Color and Morphological Parameters

Fresh weight was recorded for all sample types using a precision balance (manufacturer, model) in six replicates (*n* = 6). Flavedo colour was measured using a portable colorimeter (NH300, 3NH, Shenzhen, China), calibrated before each session with standard white and black reference tiles. Three measurements were taken per fruit at equidistant points along the equatorial region, avoiding areas affected by visible defects, sunburn, or mechanical damage, and averaged to obtain a single L*, a*, and b* value per fruit. Colour coordinates were expressed in the CIELAB colour space, where L* represents lightness, a* the green–red axis, and b* the blue–yellow axis. Morphometric analyses were conducted using a flatbed scanner (EPSON Perfection V39, Epson, Suwa, Japan). Whole fruits, equatorial fruit cross-sections, and manually separated juice vesicles were scanned against a black background with a metric scale for calibration. Images were processed using Tomato Analyzer 4.0 software. The measured parameters for fruit cross-sections were perimeter (P, cm), area (A, cm^2^), length (W, cm), height (H, cm), longitudinal diameter (W_mid, cm), and transverse diameter (H_mid, cm), whereas projected area (cm^2^) was determined for juice vesicles.

#### 2.2.2. Total Soluble Solids (TSS) and Sugar Profile Analysis

To evaluate TSS concentration, each sample (*n* = 3) were cut and squeezed in order to collect few drops of juice; then TSS levels were measured with a refractometer (Tr Turoni, 53000C, Forlì, Italy) and expressed as °Brix. Sugar characterization was determined by high-performance liquid chromatography (HPLC) using an Azura system (Knauer, Berlin, Germany) equipped with a refractive index detector (RID 2.1L, Knauer) and an Eurospher II 100-3 NH2 column (150 × 4 mm, Knauer). For each sample, 0.2 mL of juice obtained by pressing a fraction of the pulp was collected in triplicate from all five samples, filtered through a 0.45 µm membrane, and diluted with the mobile phase prior to injection. Separation was performed under isocratic conditions using acetonitrile: water (80:20, *v*/*v*) as the mobile phase, at a flow rate of 1.0 mL/min and a column temperature of 35 °C. Sugars were identified by comparison with authenticated reference standards and quantified using external calibration curves.

#### 2.2.3. Titratable Acidity

Titratable acidity (TA) was determined by titration on filtered juice samples. For each sample (*n* = 3), 2 mL of juice were diluted with approximately 100 mL of distilled water and titrated against 0.1 M NaOH using phenolphthalein (1% solution) as indicator. The endpoint was identified at the first persistent pink-violet color change. Results were expressed as grams of citric acid per 100 mL of juice, calculated from the molar quantity of NaOH consumed at the equivalence point. The TSS/TA ratio was calculated using the mean TSS and TA values obtained for each cultivar, as individual TSS and TA measurements were performed on different sample aliquots.

### 2.3. Secondary Metabolites

#### 2.3.1. Histochemical Localization of Terpenoids and Flavonoids by Confocal Microscopy

To investigate the variations in secondary metabolites, specifically terpenoids, across different genotypes and ripening stages, cross-sections of the tissue were prepared. Three fruits per genotype, including both the epicarp and pulp vesicles, were sectioned at a thickness of approximately 100 µm using an OTS 500 Oscillating Tissue Slicer (Hatfield, PA, USA). One sections for each fruit (*n* = 3) was then subjected to Nadi histochemical staining for the detection of terpenic substances, following the method described by David and Carde (1964) [22]. The staining solution consisted of 0.5 mL of 1% α-Naftolo in 40% ethanol, 0.5 mL of 1% dimethyl-p-phenylenediamine hydrochloride in water, and 49 mL of 0.05 M phosphate buffer (pH 7.2). Sections were incubated in this solution in the dark for 40 min, followed by a washing step in distilled water. The same sections were also used to assess flavonoid distribution, based on the natural autofluorescence of these compounds. Imaging of both the Nadi-stained terpenoids and the auto fluorescent flavonoids was performed using a Leica TCS SP5 (Leica Microsystems, Wetzlar, Germany) confocal laser scanning microscope. The area of the signal from terpenoids and flavonoids was quantifies using the software ImageJ (version 1.53k; National Institutes of Health, Bethesda, MD, USA) and FIJI package.

#### 2.3.2. Total Phenolic Content

Total phenolic content (TPC) in the pericarp and pulp of each genotype was determined using the Folin–Ciocalteu colorimetric assay, following the method described by Vashisth et al. (2011) [23], and expressed as mg gallic acid equivalents per kg fresh weight (mg GAE/kg FW). All measurements were performed in triplicate (*n* = 3) per genotype and tissue fraction.

#### 2.3.3. Determination of Volatile Organic Compound Profile

Volatile organic compounds (VOCs) were analysed using a PTR-ToF 8000 mass spectrometer (IONICON Analytik GmbH, Innsbruck, Austria) operated with H_3_O^+^ as the reagent ion for proton-transfer reactions. This technique enables rapid, non-destructive, and highly sensitive VOC profiling without prior sample preparation [24]. To characterize the aroma, VOC measurements were performed on fruits cut longitudinally into two halves. To ensure comparability of VOC profiles across cultivars despite differences in fruit mass, all data were normalized to a standard fruit weight of 100 g. In this configuration, both peel and pulp contributed to the headspace composition. Juice vesicles and peel were manually separated from three individual fruits per genotype and placed separately in 0.75-L glass jars, resulting in three independent biological replicates (*n* = 3) for headspace VOC analysis by PTR-ToF-MS. Jars were fitted with two Teflon tubes (0.5 mm i.d.) serving as air inlet and outlet. The inlet was connected to a zero-air generator, whereas the outlet was connected directly to the PTR-ToF-MS for dynamic headspace analysis. Prior to each measurement, an empty jar was analysed as a blank and used for background subtraction. The PTR-ToF-MS was operated at a drift tube voltage of 600 V, drift pressure of 2.2 mbar, drift gas flow of 40 sccm, and E/N ratio of 145 Td. Mass spectra were acquired over the *m*/*z* range 20–220. The inlet line and drift tube were both maintained at 100 °C to minimise condensation and adsorption losses of volatile compounds. Spectra were internally calibrated offline using the signals at *m*/*z* 29.9974 (NO^+^), 59.049 (C_3_H_7_O^+^), and 137.132 (C_10_H_17_^+^). Mass calibration was performed according to the three-point procedure described by Cappellin et al. (2010) [25], ensuring accurate mass assignment across the analysed mass range. Tentative compound identification was subsequently achieved by combining the molecular formulae generated by the PTR-ToF-MS software with published literature data [26,27,28,29].

### 2.4. Sensory Evaluation

A sensory evaluation was performed to characterize the organoleptic properties of finger lime and Faustrime fruits, with the aim of describing and comparing the distinctive sensory characteristics of each analysed genotype. The assessment was conducted by a trained panel consisting of ten assessors (five males and five females, aged 25–50 years) from the Department of Agriculture, Food, Environment and Forestry (DAGRI). All panellists had previous experience in the sensory evaluation of citrus fruits, although none had prior familiarity with finger lime or Faustrime. The sensory attributes were selected based on the descriptors proposed by Bowman et al. (2019) [30], with minor modifications to better reflect the characteristics of the studied fruits. The panel evaluated the following attributes: visual appearance, juiciness, crunchiness, popping effect in the mouth, sweetness, sourness, bitterness, citrus aroma, green aroma, minty/fresh aroma, citronella aroma, piney aroma, and intensity of the aroma perceived immediately after cutting the fruit. Each attribute was scored using a nine-point scale, where 1 = extremely poor, 3 = poor, 5 = acceptable (marketability threshold), 7 = good, and 9 = excellent. For each genotype and sampling time, five fruits were presented to the panellists for visual evaluation. Subsequently, these fruits, together with the remaining four fruits, were cut open and immediately served in plastic cups labelled with randomized identification codes. Samples were presented in random order. Between samples, panellists rinsed their mouths with water to minimise carry-over effects and residual sensory perceptions. Differences between means were performed using the least significant difference test, with probability error α = 0.05 [31].

### 2.5. Statistical Analysis

The statistical analyses were performed using GraphPad Prism 9 (GraphPad Software, San Diego, CA, USA). Data are expressed as mean ± standard deviation (SD). Differences among genotypes were assessed by one-way ANOVA followed by Tukey’s post-hoc test. Different letters indicate statistically significant differences (*p* < 0.05) among genotypes. Hierarchical clustering and PERMANOVA were performed in R (version 4.5.3 package vegan), standardized as z scores, with a small fraction of missing values imputed using the genotype mean. Clustering was based on Euclidean distance with average linkage (UPGMA), while genotype-level separation was tested by PERMANOVA (999 permutations).

## 3. Results

### 3.1. Morphological Identity

**Figure 1:**
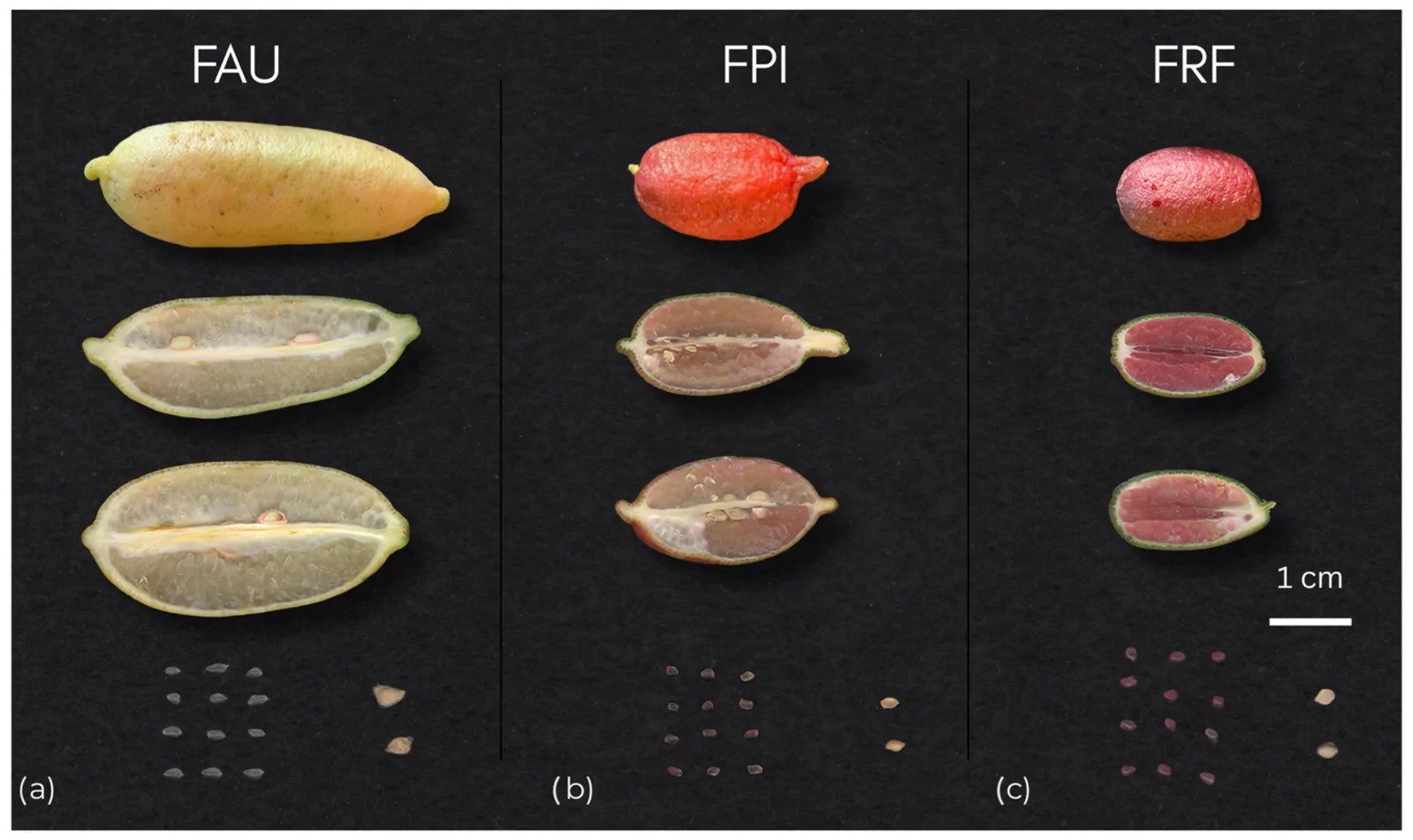
Whole fruits, fruits section, vesicles and seeds. (**a**) FAU (Faustrime), (**b**) and FPI (Pink Ice) and (**c**) FRF (Rainforest Pearl).

Morphological analyses revealed clear genotype-dependent differences among the three citrus accessions (Table 1). Fruit size varied significantly among genotypes, following the consistent order Faustrime > Pink Ice > Rainforest Pearl. Faustrime produced the largest fruits, with a mean cross-sectional area of 17.42 ± 1.13 cm^2^, almost twice that of Pink Ice (9.65 ± 0.50 cm^2^) and nearly three times that of Rainforest Pearl (6.42 ± 0.37 cm^2^). The same trend was observed for all linear descriptors, including perimeter, length, longitudinal diameter and transverse diameter (Table 1). Fresh weight closely mirrored fruit dimensions, with Faustrime being significantly heavier (30.95 ± 4.06 g) than Pink Ice (15.00 ± 2.65 g) and Rainforest Pearl (8.94 ± 1.18 g). Seed area followed a similar pattern, with significantly larger seeds in Faustrime (0.38 ± 0.08 cm^2^) than in the two finger lime cultivars. In contrast, juice vesicle size varied less markedly among genotypes. Faustrime exhibited the largest vesicles (0.114 ± 0.019 cm^2^), Pink Ice the smallest (0.088 ± 0.012 cm^2^), whereas Rainforest Pearl showed intermediate values (0.100 ± 0.011 cm^2^) that did not differ significantly from either genotype. Flavedo colour further discriminated the three accessions (Figure 1).

Faustrime displayed the highest lightness (L* = 70.68 ± 1.09) and yellowness (b* = 47.79 ± 1.65), reflecting its characteristic bright yellow peel, whereas both finger lime cultivars exhibited darker flavedos (L* ≈ 33–34) (Figure 1). Pink Ice was clearly distinguished by the highest positive a* value (16.26 ± 0.19), confirming its characteristic red-pink pigmentation, while Rainforest Pearl showed a significantly lower red component (a* = 7.18 ± 1.20).

### 3.2. Nutritional Quality

#### 3.2.1. Total Soluble Solids (TSS), Titratable Acidity (TA), Sugar Profile Analysis and Total Phenolic Content

Marked differences were also observed in fruit chemical composition. Total soluble solids ranged from 10.1 ± 0.6 °Brix in FAU to 17.8 ± 0.2 °Brix and 19.2 ± 1.4 °Brix in FPI and FRF, respectively (Table 2). HPLC analysis confirmed this pattern, revealing the highest concentrations of fructose (17.10 g L^−1^) and glucose (20.81 g L^−1^) FRF, whereas FPI exhibited intermediate concentrations and the highest sucrose content (3.84 g L^−1^). FAU consistently showed the lowest concentrations of all three soluble sugars (Table 2). Titratable acidity followed a different trend, reaching its highest value in FPI (11.56% citric acid), followed by FAU (9.38%) and FRF (8.93%) (Table 2). The TSS/TA ratio, calculated as an index of ripening stage, was lowest in FAU (1.076), intermediate in FPI (1.54), and highest in FRF (2.15), reflecting differences in the balance between sugars and acids among the three accessions. The phenolic profile (Table 2) revealed a tissue- and genotype-dependent distribution. In the pericarp, all genotypes showed high phenolic concentrations with no statistically significant differences among them (1039.99–1690.91 mg EAG/kg FW), FRF and FPI presenting the highest mean values. In the pulp, significant differences emerged: FPI exhibited the highest phenolic content (919.09 ± 47.70 mg EAG/kg FW, letter b), significantly higher than FAU (503.06 ± 22.29 mg EAG/kg FW), with FRF intermediate and not statistically distinct from either (694.88 ± 143.33 mg EAG/kg FW). The high phenolic content of the FPI pulp is particularly relevant from a nutritional standpoint, since the juice vesicles constitute the fraction directly consumed in gastronomic preparations, conferring on this cultivar a functional value that complements its chromatic appeal.

#### 3.2.2. Histochemical Localization of Terpenoids and Flavonoids

**Figure 2:**
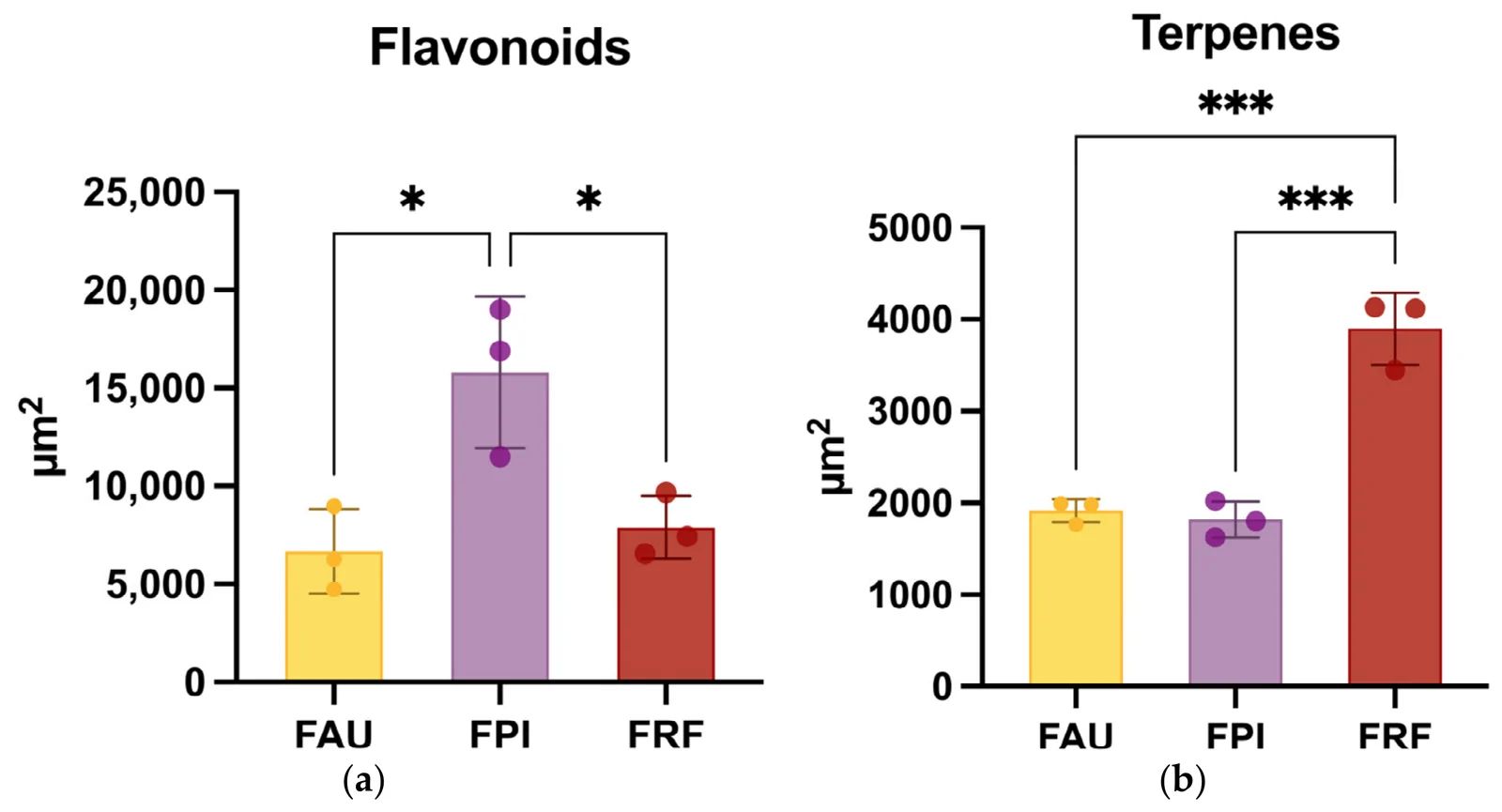
(**a**) Total area (μm^2^) of autofluorescence of flavonoids and (**b**) total area (μm^2^) of Nadi-positive secretory structures detected by confocal laser scanning microscopy for Terpens FAU (Faustrime), FRF (Rainforest Pearl) and FPI (Pink Ice). Values represent the cumulative area positive for fluorescence per section. Data were analysed using one way ANOVA followed by Tukey’s post hoc test (*p* < 0.05). Bars represent mean ± SD; *n* = 3. Asterisks indicate statistically significant differences (* *p* < 0.05; *** *p* < 0.001).

According to the area measured by confocal laser scanning microscopy (Figure 2), significant differences among the three genotypes were found. FRF showed the highest value (3899.2 ± 391.3 μm^2^), while FAU (1912.6 ± 125.6 μm^2^) and FPI (1817.5 ± 196.9 μm^2^) presented comparable, significantly lower values. Flavonoid distribution, assessed by autofluorescence, followed a markedly different pattern (Figure 2). FPI exhibited the highest area of flavonoid accumulation (15,793.81 ± 3866.92 μm^2^), which was significantly greater than both FAU (6661.32 ± 2143.57 μm^2^) and FRF (7889.81 ± 1598.80 μm^2^). The FRF and FPI genotypes did not differ significantly from one another. This contrasts with the terpenoid distribution, where FRF was the richest genotype; FPI, by contrast, accumulated the highest flavonoid concentration but showed the lowest terpenoid storage.

### 3.3. Integrated Morphometric and Physicochemical Profile

**Figure 3:**
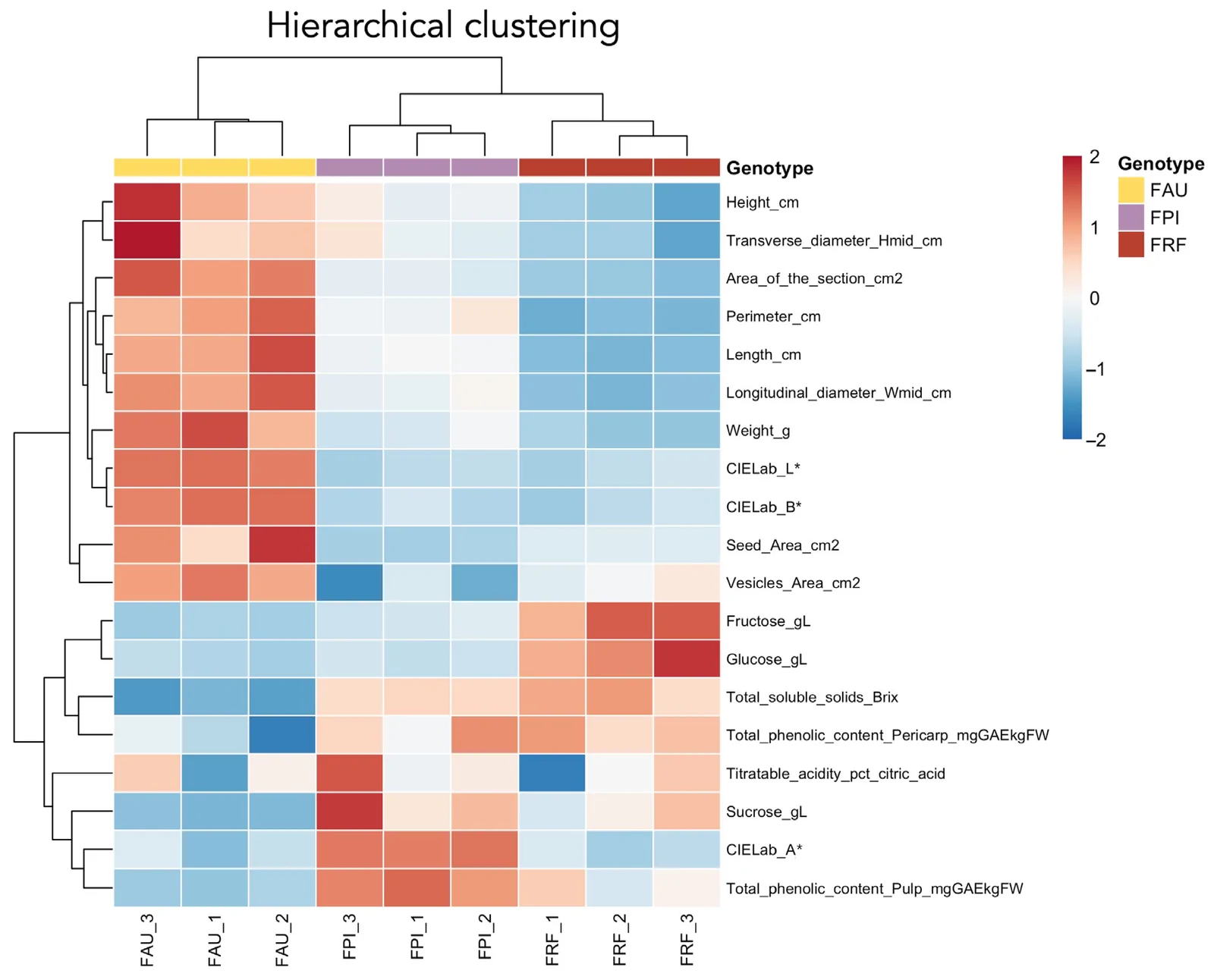
The heatmap displays z-score standardized morphometric and physicochemical traits for three genotypes: FAU (Faustrime), FPI (Pink Ice), and FRF (Rainforest Pearl). Both variables (rows) and pseudo-replicates (columns, three per genotype) were ordered by hierarchical clustering (Euclidean distance, average linkage). The color scale indicates values above the mean in red and values below the mean in blue for each variable. PERMANOVA analysis revealed a significant difference among genotypes (R^2^ = 0.874, *p* = 0.004, 999 permutations). The * symbol is the name of the variables (CIELab colorimetric parameters (L*); CIELab colorimetric parameters (A*); CIELab colorimetric parameters (B*).

To formally test whether the morphometric and physicochemical profiles differed among genotypes, a permutational multivariate analysis of variance (PERMANOVA; Euclidean distance, 999 permutations) was performed on the full set of z-score standardized variables (Figure 3). Genotype explained a large and significant proportion of the multivariate variance (R^2^ = 0.87362, F(2,6) = 20.7376, *p* = 0.004), confirming the clear genotype-level separation already visible in the hierarchical clustering (Figure 3). In the dendrogram, the two finger lime cultivars, Pink Ice and Rainforest Pearl, grouped as the most similar pair, whereas Faustrime formed a clearly distinct cluster. This pattern was driven mainly by fruit size and peel colour: FAU showed markedly higher CIELab lightness (L*) and yellowness (b*), together with larger fruit dimensions, than either finger lime cultivar. The two finger lime cultivars were mainly distinguished by their sugar and soluble solids content, with FRF showing higher total soluble solids and fructose levels compared to FPI. Since the values of six of the nineteen variables were averaged in pairs to generate three pseudoreplicates per genotype, the precise values of R^2^ and *p* should be interpreted with caution. Averaging replicates in this way tends to reduce variability inside each genotype. Even so, the clear distinction between the different genotypes remained consistent across all variables, confirming the strong reliability and robustness of the multivariate test results.

### 3.4. Aroma Fingerprint

#### 3.4.1. Volatile Organic Compound Profile

The volatile organic compound (VOC) emission of the three cultivars was characterised by PTR-ToF-MS, and 36 mass peaks above the detection threshold were tentatively identified (Table 3). The total VOC emission differed markedly among genotypes, FRF showed the highest total emission (2048.08 ppbv), approximately twice that of FPI (1118.76 ppbv) and more than four times that of FAU (480.34 ppbv). The same order was observed for the total terpene emission, which reached 1629.07 ppbv in FRF, 787.22 ppbv in FPI, and 247.80 ppbv in FAU. The number of detected signals was comparable across the three genotypes (30 in FRF, 32 in FPI, and 31 in FAU). In all three genotypes, the volatile profile was dominated by terpene-related ions. The base peak was detected at *m*/*z* 81.069 (C_6_H_9_^+^), which is the major fragment generated by protonated monoterpenes in PTR-ToF-MS, particularly from the molecular ion detected at *m*/*z* 137.132 (C_10_H_17_^+^) [19]. Accordingly, both ions followed the same emission pattern (Rainforest Pearl > Pink Ice > Faustrime), indicating a markedly higher abundance of monoterpenes in Rainforest Pearl. Additional terpene-related fragments, including *m*/*z* 95.086 and *m*/*z* 93.069, showed the same genotype-dependent trend, further supporting the predominance of terpene metabolism in the volatile profile of the two finger lime cultivars (Table 3). Several additional oxygenated terpenes (e.g., *m*/*z* 153.127) and sesquiterpene-related ions (e.g., *m*/*z* 149.132, 205.195), were detected only at relatively low abundances across all cultivars. Among the non-terpene compounds, acetone (*m*/*z* 59.049) was the most abundant oxygenated volatile in all genotypes, whereas acetic acid (*m*/*z* 61.027) reached its highest abundance in Pink Ice. Benzene (*m*/*z* 79.054) was particularly abundant in FRF, while methanol (*m*/*z* 33.033) showed similar emissions in FPI and FAU and lower levels in Rainforest Pearl. FPI was the only genotype exhibiting a detectable signal at *m*/*z* 103.075, tentatively assigned to butyl formate, an ester characterized by fruity, plum-like aroma and sweetness, and previously reported as a natural trace aroma constituent of other fruit profiles [32].

#### 3.4.2. Sensory Profile

**Figure 4:**
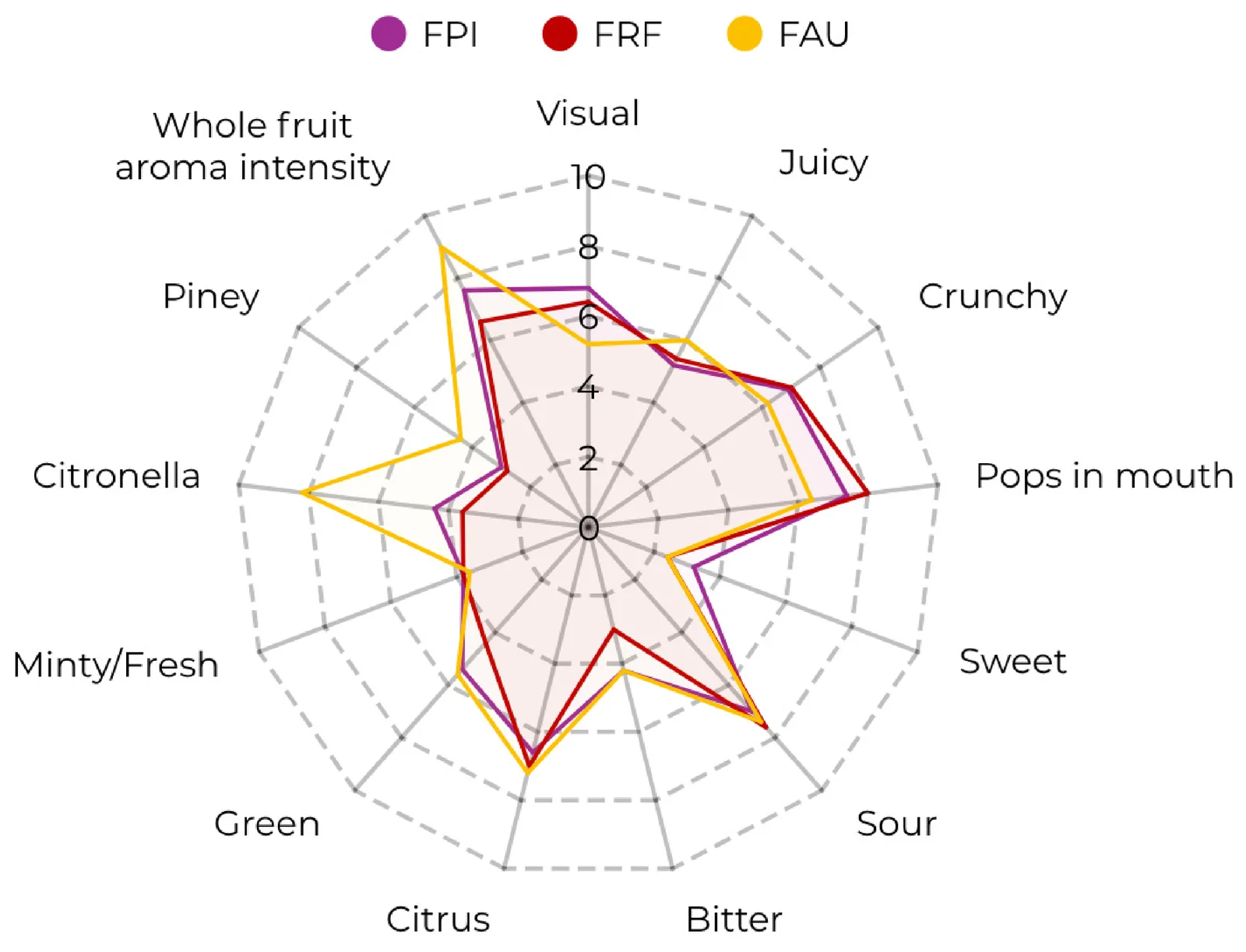
Sensory profile of FPI (Pink Ice). FRF (Rainforest Pearl). and Faustrime (FAU) assessed by a mixed panel of 10 trained experts. Scores are on a nine-point hedonic scale (1 = extremely poor; 5 = acceptability threshold; 9 = excellent). Attributes: Visual, Juicy, Crunchy, Pops in mouth, Sweet, Sour, Bitter, Citrus, Green, Minty/Fresh, Citronella, Piney, Whole-fruit aroma intensity.

Sensory evaluation by the trained panel distinguished the three genotypes across visual, textural, gustatory, and olfactory attributes (Figure 4). Visually, FPI achieved the highest score (6.8), reflecting the immediate attractiveness of its red-pink pericarp, followed by FRF (6.4) and FAU (5.2). For the textural attribute “pops in mouth”, which describes the characteristic bursting sensation of citrus caviar vesicles, FRF scored highest (8.0), followed by FPI (7.4) and FAU (6.4). This trend is consistent with the smaller, more turgid vesicles of the pure finger lime cultivars, which are associated with higher internal osmotic pressure. In the flavour domain, all genotypes were dominated by sourness (FRF 7.6, FAU 7.4, FPI 7.0) and exhibited uniformly low sweetness (FPI 3.2; FRF and FAU 2.4), in agreement with the high titratable acidity and low sugar contents determined analytically. The most distinctive olfactory attribute was citronella, for which Faustrime scored markedly higher (8.2) than the pure cultivars (FPI 4.4, FRF 3.6), together with the highest overall aroma intensity of the whole fruit (9.0, compared with 7.6 for FPI and 6.6 for FRF). Overall, these results identify Faustrime as the most aromatically distinctive genotype, whereas the pure finger lime cultivars, particularly Rainforest Pearl, were characterised by superior vesicle texture and greater visual appeal.

## 4. Discussion

This study demonstrates that a combined analytical approach integrating morphometric, physicochemical, histochemical, volatile, and sensory analyses delineates a cultivar-specific fruit quality profile that clearly distinguishes finger lime and Faustrime varieties. The results derived from this multiparametric framework effectively differentiated the unique profile of each genotype. These findings support earlier research demonstrating that citrus fruit quality is jointly dictated by genotype, tissue type, and environmental factors [12,33]. The clear size difference observed in Faustrime compared with the two pure *Citrus australasica* varieties, as evidenced by all morphological parameters and fresh weight measurements, may reflect, at least in part, its distinct hybrid genetic background. Faustrime originates from a cross involving *C. australasica*, *C. aurantiifolia*, and *Fortunella japonica*, and the contribution of parental genomes to fruit size is a recognized feature of citrus breeding, where fruit weight and size are inherited traits influenced by genomic hybridization [34]. In contrast, the comparatively smaller differences observed in juice vesicle area suggest that vesicle morphology may be a more conserved trait than overall fruit size. This observation is of practical importance, because vesicle size and attributes are a key descriptors of the “citrus caviar” product and contribute substantially to its visual quality and appreciability. The sensory panel addressed this attribute directly, scoring the textural descriptor “pops in mouth” highest in FRF, followed by FPI and FAU. The ranking is the reverse of vesicle size, since FAU showed the largest vesicles and FPI the smallest: the bursting sensation therefore appears to depend on the turgidity of the vesicles rather than on their dimensions [35]. Flavedo colour emerged as one of the most discriminating traits among the three accessions. The strong red pigmentation of FPI, reflected by its highest a* value (16.26 ± 0.19), indicates a high anthocyanin accumulation in the peel. The histochemical data support this interpretation independently, since Pink Ice also showed the largest area of autofluorescent flavonoids in the flavedo (15,793.81 ± 3866.92 µm^2^, twice the value measured in Rainforest Pearl and more than twice that of Faustrime). Anthocyanins belong to the flavonoid class, and their accumulation in the peel of pigmented citrus is strongly genotype dependent. In contrast, Rainforest Pearl (*C. australasica* var. sanguinea) and the Faustrime displayed distinct colour identities. Peel and pulp colour are consolidated descriptors in the varietal classification of finger. The results obtained on pigmentation observed here are consistent with previous evidence showing that while genetics dictates the potential for pigment synthesis, its full expression depends heavily on local pedoclimatic conditions [36,37]. Given that peel color is the first quality attribute consumers notice and a major driver in purchasing exotic fruits, these distinct color profiles directly influence how each variety can be positioned in the market [15]. Here too the instrumental and the sensory descriptions converged, Pink Ice obtaining the highest visual score from the panel (6.8), ahead of Rainforest Pearl (6.4) and Faustrime (5.2). All three cultivars exhibited high titratable acidity and relatively low sugar content. This characteristic profile is typical of finger limes and their hybrids, whose culinary appeal relies primarily on their intense, aromatic acidity rather than on sweetness [38]. The panel scores reproduce the analytical determinations closely: sourness dominated in all three genotypes (Rainforest Pearl 7.6, Faustrime 7.4, Pink Ice 7.0) and sweetness remained uniformly low (Pink Ice 3.2; Rainforest Pearl and Faustrime 2.4). FRF combined the highest sugar levels (fructose 17.10 ± 2.45 g/L, glucose 20.81 ± 4.03 g/L) with the lowest titratable acidity (8.93%), achieving the most balanced sugar-to-acid ratio. Meanwhile, FPI presented the most acidic profile (11.56%), though titratable acidity did not differ significantly among the three genotypes. Phenolic profiling identified FPI as the richest accession within the edible pulp fraction (919.09 ± 47.70 mg GAE/kg FW). This phenolic distribution closely aligns with the pattern previously reported for finger lime by Wang et al. [12], who demonstrated that the phenolic profile of *C. australasica* is strongly dictated by both cultivar and tissue type. Likewise, Lim et al. [39] reported substantial differences among cultivars in total phenolic content and antioxidant capacity. The Folin assay on the pulp and the measurement of autofluorescent flavonoid areas in the flavedo showed the highest values in Pink Ice, followed by Rainforest Pearl, and lastly Faustrime. The two independent approaches showed a consistent trend, with Pink Ice exhibiting both the highest total phenolic content in the pulp and the largest autofluorescent flavonoid area in the flavedo, highlighting a distinctive phenolic profile under the conditions of the present study. The high phenolic content found in FPI pulp holds significant nutritional value, given that juice vesicles are the primary portion consumed in culinary uses. These findings reinforce the potential of this cultivar as a specialty niche ingredient as well as a functional food [1]. Histochemical analysis identified Rainforest Pearl as the genotype showing the highest accumulation of terpenoids within the secretory structures of the flavedo. In citrus fruits, the flavedo represents the principal site of terpenoid biosynthesis and storage, where essential oils accumulate within schizolysigenous secretory cavities before volatile terpenes are released into the atmosphere [40]. Flavonoids, quantified on the same sections, delineated a second and independent axis of variation. Pink Ice, which showed the lowest terpenoid accumulation of the three accessions, showed the highest flavonoid accumulation, whereas Rainforest Pearl combined the highest terpenoid accumulation with intermediate flavonoid values (Figure 2). The two classes of secondary metabolites therefore vary independently of one another in this germplasm and contribute to different quality attributes, terpenoids to aroma and flavonoids to pigmentation and to the antioxidant properties of the tissue. The PTR-ToF-MS headspace analysis closely mirrored the trends observed with terpenoid staining. Rainforest Pearl exhibited the highest total emission of both volatile organic compounds and terpenes (1629.07 ppbv of terpenes, compared to 787.22 ppbv in Pink Ice and 247.80 ppbv in Faustrime). Peel storage capacity and headspace release are therefore mutually consistent in this cultivar, confirming the suitability of PTR-ToF-MS for the characterization of citrus accessions based on their volatile emissions. The sensory data describe a different ordering. Faustrime, whose total terpene emission was more than six times lower than that of Rainforest Pearl, obtained the highest scores both for the citronella descriptor and for overall whole-fruit aroma intensity (Figure 4). Two independent olfactory attributes, scored separately by the panel, thus ranked the genotypes in the reverse order to emitted terpene mass. The quantity of terpenes accumulated in peel tissues and the fraction emitted into the headspace are conceptually distinct from the fraction perceived during consumption; considering these descriptors as complementary rather than equivalent therefore provides a more comprehensive understanding of terpene characteristics among the different accessions. Aroma perception depends not only on the overall intensity of volatile emissions, but also on the qualitative composition of the volatile blend. Faustrime’s distinct chemotype marked by limonene, β-phellandrene, γ-terpinene, and a notable citronellal contribution matches previous reports from Italian-grown fruit [19] and explains the strong citronella note recorded by our sensory panel. Despite limonene dominating the volatile profile in all finger lime cultivars, each genotype retains a unique aromatic identity [13]. This confirms that aroma distinctiveness in finger lime and its hybrids is driven by the relative ratios of specific volatiles rather than simply the presence of major terpenes. These results reinforce this interpretation, as the genotype with the lowest total emissions was judged the most aromatically unique. Together, these observations highlight how instrumental analysis and sensory profiling complement one another. Histochemical staining and PTR-ToF-MS reliably measure terpenoid reserves and headspace release, showing high consistency (Figure 2). Meanwhile, the sensory panel evaluates the consumer experience, responding to blend the composition of the volatile blend rather than its overall concentration. Both approaches are thus critical for assessing aroma quality in specialty citrus, and neither can replace the other in cultivar selection. Beyond their biological significance, these integrated quality fingerprints also have important practical implications for the commercial valorisation of the investigated genotypes. Our findings indicate that finger lime cultivars and Faustrime occupy complementary rather than competing positions within the premium specialty citrus market. The two *C. australasica* cultivars are characterised by the pigmentation, vesicle texture, and phenolic richness typically associated with the “citrus caviar” concept, with Pink Ice distinguished by its elevated phenolic content, in the pulp as well as in the flavedo, and Rainforest Pearl by its more balanced sweet to sour profile and higher volatile emissions. In contrast, Faustrime combines larger fruit size with a distinctive aromatic identity, highlighting its potential for specialty citrus markets where aroma represents a key quality attribute. This complementary nature reinforces the co-cultivation of finger lime varieties and Faustrime as a versatile portfolio of high-value specialty citrus for the expanding Mediterranean market [16,18,19]. Overall, these results show that the differences among Pink Ice, Rainforest Pearl, and Faustrime aren’t limited to just one type of trait. The multivariate analysis provides clear statistical evidence for this (Figure 3). PERMANOVA found a significant effect of genotype when looking at all the morphometric and physicochemical traits together. This means the three accessions are distinguished by a consistent pattern across several traits, not just by differences in individual variables.

## 5. Conclusions

This study provides the first integrated fruit quality fingerprint of two finger lime cultivars, ‘Pink Ice’ and ‘Rainforest Pearl’, together with the trigeneric hybrid Faustrime cultivated in a Mediterranean region, establishing a comprehensive quality baseline for these emerging specialty citrus fruits. By integrating morphometric, physicochemical, histochemical, volatilomic, and sensory analyses, the study demonstrated that the three accessions possess distinct and complementary quality profiles, clearly differentiating them in terms of fruit morphology, chemical composition, aroma, and sensory characteristics. Each genotype exhibited attributes that support different commercial applications. Pink Ice was distinguished by its intense pigmentation and phenolic-rich pulp, Rainforest Pearl by its higher sugar content, balanced sweet/sour profile, and elevated terpene emissions, whereas Faustrime combined larger fruit size with a distinctive citronella aroma, highlighting its unique aromatic identity. The integrated quality fingerprint proposed here provides a robust framework for the characterization, comparison, and commercial valorization of specialty citrus germplasm beyond finger lime, supporting genotype-specific cultivar selection and market positioning. Rather than representing competing products, these accessions should be regarded as complementary components of a diversified portfolio of premium specialty citrus, capable of satisfying different consumer preferences and market demands. Although the present work was conducted at a single production site, during one growing season and at a single harvest stage, it provides a solid foundation for future research. Multi-year and multi-site investigations, including different ripening stages and complemented by shelf-life studies, consumer acceptance tests, and comprehensive volatile compound identification, will be essential to validate these findings and support evidence-based recommendations for cultivar selection, harvest management, and product development.

## Figures and Tables

**Table 1:** 1 Morphological, dimensional, and CIELab colorimetric parameters of fruit. Data were analysed using one-way ANOVA followed by Tukey’s post-hoc test; different letters indicate statistically significant differences among genotypes (*p* < 0.05). Values represent mean ± SD, *n* = 6. Trasversal section of the fruits, FAU (Faustrime), FRF (Rainforest Pearl) and FPI (Pink Ice).

		FAU	FPI	FRF
Fruit	Area of the section (cm^2^)	17.42 ± 1.13 a	9.65 ± 0.50 b	6.42 ± 0.37 c
	Weight (g)	30.95 ± 4.06 a	15.00 ± 2.65 b	8.94 ± 1.18 b
	Perimeter (cm)	18.2 ± 0.8 a	13.9 ± 0.3 b	10.7 ± 0.4 c
	Length (cm)	7.4 ± 0.5 a	5.5 ± 0.1 b	3.9 ± 0.1 c
	Height (cm)	3.1 ± 0.3 a	2.5 ± 0.1 b	2.1 ± 0.1 c
	Longitudinal diameter (W_mid) (cm)	7.1 ± 0.4 a	5.2 ± 0.3 b	3.9 ± 0.1 c
	Transverse diameter (H_mid) (cm)	3.0 ± 0.3 a	2.5 ± 0.2 b	2.0 ± 0.1 c
	CIELab colorimetric parameters (L*)	70.68 ± 1.09 a	33.23 ± 1.77 b	34.30 ± 3.14 b
	CIELab colorimetric parameters (A*)	7.06 ± 1.67 a	16.26 ± 0.19 a	7.18 ± 1.20 b
	CIELab colorimetric parameters (B*)	47.79 ± 1.65 a	11.16 ± 3.47 b	9.73 ± 3.87 b
Seed	Area (cm^2^)	0.38 ± 0.08 a	0.15 ± 0.01 b	0.21 ± 0.01 b
Vesicles	Area (cm^2^)	0.11 ± 0.02 a	0.09 ± 0.01 b	0.10 ± 0.01 ab

**Table 2:** Total soluble solids (TSS), sugar profile, titratable acidity (TA), and total phenolic content (TPC). TSS is expressed as °Brix; sugar composition; TA was determined by titration and expressed as mL of 0.1 M NaOH consumed and as % citric acid (g/100 mL); TSS/TA ratio (ripening index); TPC separately in the pericarp and pulp. FAU (Faustrime), FRF (Rainforest Pearl) and FPI (Pink Ice). Data were analysed using one-way ANOVA followed by Tukey’s post-hoc test; different letters within the same row indicate statistically significant differences among genotypes (*p* < 0.05). *n* = 3.

			FAU	FPI	FRF
Total soluble solids		°Brix	10.1 ± 0.6 a	17.8 ± 0.2 b	19.2 ± 1.4 b
Sugar profile	Fructose	g/L	1.97 ± 0.42 a	5.00 ± 0.89 b	17.10 ± 2.45 c
	Glucose	g/L	3.18 ± 0.99 a	4.94 ± 0.67 a	20.81 ± 4.03 b
	Sucrose	g/L	0.65 ± 0.08 a	3.84 ± 1.14 b	2.61 ± 0.91 b
Titratable acidity		% citric acid	9.38 ± 3.1 a	11.56 ± 2.67 a	8.93 ± 3.6 a
TSS/TA			1.076	1.54	2.15
Total phenolic content	Pericarp	mg GAE/kg FW	1039.99 ± 315.22 a	1606.12 ± 332.26 a	1690.91 ± 171.35 a
	Pulp	mg GAE/kg FW	503.06 ± 22.29 a	919.09 ± 47.70 b	694.88 ± 143.33 ab

**Table 3:** Volatile organic compounds (VOCs) identified in the headspace of Faustrime (FAU), Pink Ice (FPI), and Rainforest Pearl (FRF) fruits by PTR-ToF-MS. For each mass peak, the code number, mass-to-charge ratio (*m*/*z*), chemical formula, and tentative compound identification are reported, together with the mean concentration ± SD (ppbv) for each genotype. Total VOC emission, total terpene emission, and the number of detected signals per genotype are reported at the bottom of the table.

Code Number	M/Z	Chemical Formula	Tentative Identification	FRF		FPI		FAU	
				Average	SD	Average	SD	Average	SD
1	31.018	CH_3_O^+^	Formaldehyde	2.62	1.29	5.30	1.35	1.41	0.24
2	33.033	CH_5_O^+^	methanol	6.54	2.06	18.41	5.35	19.89	2.90
3	41.040	C_3_H_5_^+^	Alkyl fragment	11.90	4.55	12.38	2.33	15.05	7.36
4	43.018	C_2_H_3_O^+^	Alkyl fragment (ethenone/incensole acetate)	39.23	6.37	42.24	10.50	9.44	0.95
5	43.054	C_3_H_7_^+^	Alkyl fragment (propene)	13.14	1.27	5.41	2.97	7.19	2.76
6	45.033	C_2_H_5_O^+^	acetaldehyde	35.26	9.26	36.60	3.50	26.39	7.98
7	55.054	C_4_H_7_^+^	C4 aldehydes fragment (butanal)	10.75	2.55	4.88	0.84	1.79	0.25
8	57.033	C_3_H_5_O^+^	Alkyl fragment (hexanal/1-butanol/1-octanol)	2.26	0.53	2.97	0.80	2.26	0.15
9	57.069	C_4_H_9_^+^	Alkyl fragment (hexanol/valeric acid)	4.85	0.56	2.20	0.23	1.01	0.25
10	59.049	C_3_H_7_O^+^	Aceton	176.36	29.47	84.07	21.96	110.90	21.07
11	61.027	C_2_H_5_O_2_^+^	Acetic acid	18.68	5.23	78.72	10.67	21.34	0.69
12	69.069	C_5_H_9_^+^	C5/isoprene	18.64	3.43	5.51	0.68	4.13	1.51
13	71.049	C_4_H_7_O^+^	Methyl vinyl ketone	11.33	2.78	6.87	0.93	5.23	2.79
14	79.054	C_6_H_7_^+^	C6 fragments	44.73	7.71	14.60	3.28	2.90	0.36
15	81.069	C_6_H_9_^+^	Terpene fragments/C6 fragments	1046.32	329.75	457.83	73.45	130.79	14.57
16	83.086	C_6_H_11_^+^	C6 compounds (e.g., hexenal fragment)	10.39	1.60	3.00	0.43	1.40	0.22
17	91.054	C_7_H_7_^+^	Monoterpene fragments	11.04	2.41	3.03	0.63	1.08	0.68
18	93.069	C_7_H_9_^+^	Terpenes fragments	44.17	3.71	16.43	5.73	9.97	1.74
19	95.086	C_7_H_11_^+^	Terpenes fragments	185.97	56.54	56.23	13.18	24.44	12.52
20	97.101	C_7_H_13_^+^	Terpenes fragments	0.65	0.16	0.85	0.19	0.00	0.00
21	99.080	C_6_H_11_O^+^	Hexenals/methyl-pentenone	0.00	0.00	0.00	0.00	0.80	0.14
22	103.075	C_5_H_11_O_2_^+^	butyl formate	0.00	0.00	4.14	2.40	0.00	0.00
23	107.086	C_8_H_11_^+^	Ethyl benzene/Terpenes fragment	12.35	4.51	4.25	2.26	0.84	0.08
24	109.101	C_8_H_13_^+^	Terpenes fragments	3.89	0.88	2.09	0.65	2.71	0.54
25	111.117	C_8_H_15_^+^	Monoterpenes fragments	1.23	0.25	0.56	0.05	0.00	0.00
26	113.096	C_7_H_13_O^+^	2-heptenal	0.00	0.00	0.00	0.00	0.58	0.09
27	119.085	C_9_H_11_^+^	Terpenes fragments	0.63	0.09	0.77	0.21	0.74	0.32
28	121.101	C_9_H_13_^+^	Terpenes fragments	9.25	3.22	2.28	0.09	1.78	0.64
29	123.116	C_9_H_15_^+^	Terpenes fragments	0.76	0.13	0.75	0.16	0.00	0.00
30	125.132	C_9_H_17_^+^	Terpenes fragments	0.00	0.00	1.29	0.41	0.00	0.00
31	135.117	C_10_H_15_^+^	p-Cymene	4.95	0.95	5.78	2.63	1.41	0.42
32	137.132	C_10_H_17_^+^	Monoterpenes	314.39	140.76	234.84	136.73	66.50	29.92
33	149.132	C_11_H_17_^+^	Sesquiterpenes fragments	0.00	0.00	0.00	0.00	1.05	0.30
34	153.127	C_10_H_17_O^+^	Terpenoid-like compound (e.g., camphor. fenchone)	3.53	2.39	2.20	1.76	1.73	0.65
35	155.143	C_10_H_19_O^+^	Alcohol monoterpenes (e.g., sabinene hydrate)	0.00	0.00	0.00	0.00	0.68	0.21
36	205.195	C_15_H_25_^+^	Sesquiterpenes	2.30	0.59	2.29	0.60	4.92	0.44
Total VOC emissions (ppbv)				2048.08		1118.76		480.34	
Total Terpene emissions (ppbv)				1629.07		787.22		247.80	
Number of signals detected				30		32		31	

## Data Availability

The original contributions presented in this study are included in the article. Further inquiries can be directed to the corresponding author.
